# Development of stable reporter system cloning *luxCDABE *genes into chromosome of *Salmonella enterica *serotypes using Tn7 transposon

**DOI:** 10.1186/1471-2180-10-197

**Published:** 2010-07-23

**Authors:** Kevin Howe, Attila Karsi, Pierre Germon, Robert W Wills, Mark L Lawrence, Richard H Bailey

**Affiliations:** 1 Department of Pathobiology and Population Medicine, College of Veterinary Medicine, Mississippi State University, Mississippi State, MS 39762, USA; 2 Department of Basic Sciences, College of Veterinary Medicine, Mississippi State University, Mississippi State, MS 39762, USA; 3 Institute for Digital Biology, Mississippi State University, Mississippi State, MS 39762, USA; 4 INRA, UR 1282 Infectiologie Animale et Santé Publique, Laboratoire de Pathogénie Bactérienne, Nouzilly, France

## Abstract

**Background:**

Salmonellosis may be a food safety problem when raw food products are mishandled and not fully cooked. In previous work, we developed bioluminescent *Salmonella enterica *serotypes using a plasmid-based reporting system that can be used for real-time monitoring of the pathogen's growth on food products in short term studies. In this study, we report the use of a Tn7-based transposon system for subcloning of *luxCDABE *genes into the chromosome of eleven *Salmonella enterica *serotypes isolated from the broiler production continuum.

**Results:**

We found that the *lux *operon is constitutively expressed from the chromosome post-transposition and the *lux *cassette is stable without external pressure, i.e. antibiotic selection, for all *Salmonella enterica *serotypes used. Bioluminescence expression is based on an active electron transport chain and is directly related with metabolic activity. This relationship was quantified by measuring bioluminescence against a temperature gradient in aqueous solution using a luminometer. In addition, bioluminescent monitoring of two serotypes confirmed that our chicken skin model has the potential to be used to evaluate pathogen mitigation strategies.

**Conclusions:**

This study demonstrated that our new stable reporting system eliminates bioluminescence variation due to plasmid instability and provides a reliable real-time experimental system to study application of preventive measures for *Salmonella *on food products in real-time for both short and long term studies.

## Background

*Salmonella enterica *is indigenous to the gastrointestinal tracts of many mammals, birds, and reptiles without the manifestation of adverse effects on the host. However, subclinical infections of *Salmonella *in animals have the potential to cause disease in humans exposed to food products that are mishandled during processing or inappropriately cooked [1,2]. Cross-contamination during the slaughter process contributes to the transmission of food borne pathogens and therefore increases the risk of disease in humans. Throughout the processing plant, opportunities arise for the spread of bacteria from contaminated carcasses to uncontaminated carcasses [3,4].

Regardless of whether the source of contamination was pre-harvest or post-harvest, *Salmonella *is difficult to remove from carcasses due to its ability to adhere to chicken skin and endure the different stages of processing [5]. Laboratory research, as well as in-plant trials, has demonstrated this relationship [6-9]. Therefore, persistence of *Salmonella *within the processing plant may be partially explained by interactions between chicken skin and *Salmonella *[10]. Under controlled conditions, chemical treatments are effective in the reduction of *Salmonella *levels on broiler carcasses or skin [11-14]. However, gaps in the knowledge base exist relative to the persistence of *Salmonella *during processing and the most appropriate methods for reduction and control of the microorganism.

Bioluminescence imaging (BLI) is a technique that can be used for real-time quantification and tracking of live bacteria in hosts [15-18]. Previously, a BLI based real-time monitoring system for *Salmonella enterica *serotypes was developed by our group that employs the plasmid pAK*lux*1, which carries a bacterial luciferase gene isolated from *Photorhabdus luminescens *[19]. However, the use of this plasmid-based bioluminescence system requires continuous antibiotic selection during the course of experiments to prevent plasmid instability in *Salmonella enterica *serotypes [19], which may not be suitable for long-term *in-vitro *and *in-vivo *studies.

In response to this limitation, we now report cloning of the *luxCDABE *operon into a stable tn7-based transposon system that inserts the *luxCDABE *genes into a specific location in the *Salmonella *chromosome. We successfully used this transposon system to stably insert the bacterial *lux *operon into eleven *Salmonella enterica *serotypes isolated from the broiler production continuum, including post hatchery, prior to harvest, arrival at the plant, pre-chill tank, and post-chill tank. We also conducted a series of experiments to quantify bioluminescence expression in these *Salmonella enterica *isolates under environmental conditions that may be present in poultry processing. This reporter system can be applied in future research to further understand how *Salmonella *are able to persist throughout the poultry processing continuum, and similar situations pertinent to the food industry.

## Results and Discussion

### Construction of plasmid pBEN276

**Figure 1:**
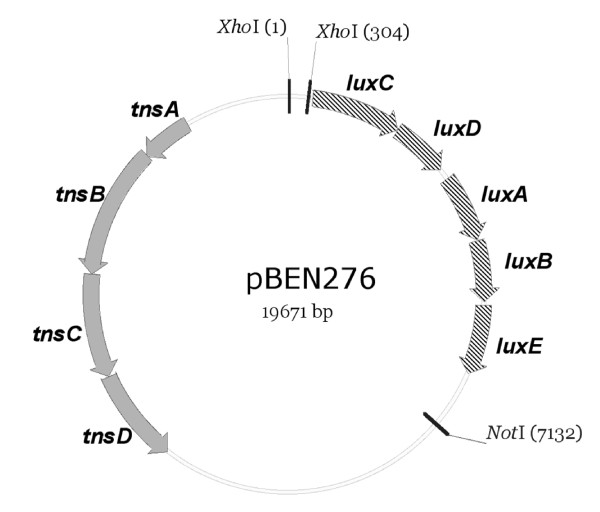
**Plasmid pBEN276 vector**. *tnsABCD *are the genes required for transposition. *luxCDABE *encodes for luciferase and is flanked by Tn7 transposon arms (vertical bars at restriction sites *Xho*I and *Not*I). The expression of *lux *genes is driven by *E. coli frr *gene promoter between the *Xho*I sites.

Plasmid pGRG25 features a site-specific recombination system based on the bacterial transposon Tn7 [20]. The Tn7 system inserts at the *attTn7 *site and is oriented specifically such that the right end of Tn7 is adjacent to the 3' end of the *glmS *gene [21], and it has been used for transgene insertion into the chromosome of *Escherichia coli*, *Salmonella*, and *Shigella *[20]. Plasmid pGRG25 is also a temperature-sensitive delivery plasmid that can be cured after transgene insertion at the *attTn7 *site by culturing at 42°C. Plasmid pBEN276 contains the *luxCDABE *operon between the Tn7 transposon arms on plasmid pGRG25, and its expression is driven by the *E. coli frr *promoter (Figure 1), which controls expression of a house-keeping gene encoding ribosome recycling factor. Thus the *lux *operon will be expressed constitutively. The chromosomal insertion point is specific and insertion of *lux *operon does not disrupt the function of *glmS *gene, therefore it is highly unlikely that bacterial physiology will be affected adversely [20].

### Characterizing the bioluminescent properties of *Salmonella enterica*

**Figure 2:**
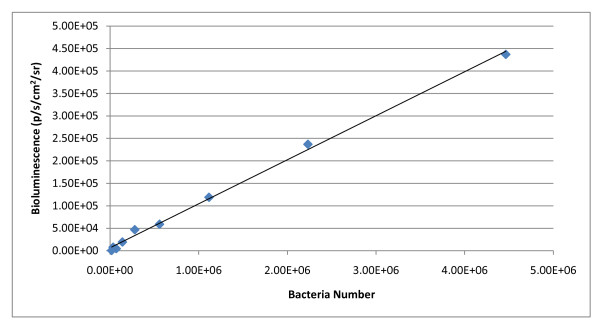
**Correlation of bioluminescence against bacterial numbers**. Plot and linear regression equation of bioluminescence flux against bacterial numbers for *S*. Montevideo. r^2 ^= 0.94, *P *= < 0.0001.

Plasmid pBEN276 was utilized to insert the bacterial *lux *operon into chromosomes of eleven *Salmonella enterica *serotypes. Bioluminescence correlated well to bacterial population density in all serotypes used, as exemplified in *S*. Montevideo (p = < 0.0001, r^2^0.94) (Figure 2). The minimum detectable concentration of all eleven serotypes was, in decreasing order (CFU/mL): *S*. Kentucky - 8.00 × 10^4^; *S*. Mbandaka - 4.99 × 10^4^; *S*. Enteritidis - 3.10 × 10^4^; *S*. Schwarzengrund - 2.78 × 10^4^; *S*. Montevideo - 1.74 × 10^4^; *S*. Alachua - 1.07 × 10^4^; *S*. Typhimurium - 6.72 × 10^3^; *S*. Seftenberg - 6.40 × 10^3^; *S*. Heidelberg - 5.28 × 10^3^; *S*. Newport - 4.64 × 10^3^; *S*. Braenderup - 4.16 × 10^3^. Minimum detectable numbers of *Salmonella *isolates expressing bioluminescence from the chromosome were higher compared to minimum detectable numbers of *Salmonella *isolates expressing plasmid-based bioluminescence [19]. One possible explanation for this difference is a copy number effect; a single copy of the *lux *operon is inserted into the chromosome with the Tn7 system, while multiple copies of the gene are expressed in plasmid systems. Plasmid pAK*lux*1 is a pBBR1 derived plasmid which characteristically has a medium copy number (~30 copies/cell) [22]. Another possible explanation is due to promoter effect; the *frr *promoter drives expression of *luxCDABE *in the Tn7 system, and the *lacZ *promoter drives expression in the pAK*lux*1 plasmid system [19]. Our previous work showed bioluminescent *Salmonella *isolates carrying plasmid pAK*lux*1 emit, on average, 6.3351 p/s/cm^2^/sr per CFU [19]; in comparison, *Salmonella *isolates carrying the *lux *operon in the chromosome emit, on average, 0.0795 p/s/cm^2^/sr per CFU. The intended purpose for this system is to use it as a screening tool for potential pathogen mitigation strategies, and this threshold of detection is sufficient for this purpose.

### Transgene stability in the chromosome of *Salmonella enterica*

**Figure 3:**
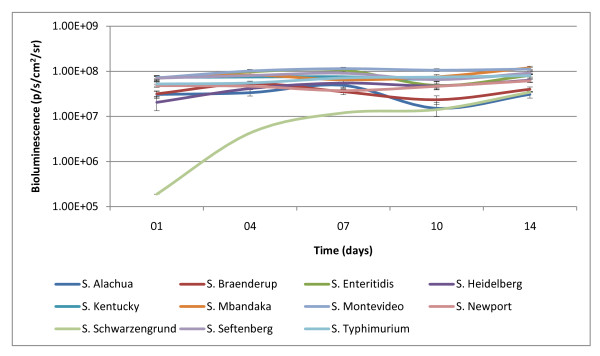
**Stability of transgene in chromosome of *Salmonella enterica *serotypes**. *Salmonella enterica *isolates carrying transgene *luxCDABE *in their chromosome were subcloned under non-selective conditions for 14 days. Bioluminescence was quantified approximately every 3 days and normalized with bacterial density (OD_600_).

Our group evaluated the stability of the *lux *operon in the chromosome following transposition by subcloning bioluminescent *Salmonella enterica *serotypes under non-selective conditions for 14 days at 37°C. Previous work from our group with plasmid-based bioluminescence expression showed the plasmid was unstable without antibiotic selection. The average half-life of plasmid pAK*lux*1, which contains the *luxCDABE *cassette, was approximately seven days in *Salmonella enterica *serotypes without antibiotic selection [19]. This current study provides evidence for a 14 day period indicating stability of the *lux *operon in the chromosome of these *Salmonella enterica *serotypes with minimal bioluminescent flux (Figure 3). A notable observation was low initial expression of bioluminescence from *S*. Schwarzengrund (10^5 ^p/s/cm^2^/sr). This serotype increased bioluminescence expression over the course of the experiment and reached similar levels of the other serotypes at approximately day 10 (10^7 ^p/s/cm^2^/sr). The differences observed for *S*. Schwarzengrund are interesting. It is important to note that the Tn7 transposon system does not insert randomly in the *Salmonella *chromosome. The Tn7 transposon system is site specific; insertion is only allowed at the *attTn7 *site. Therefore, '*luxCDABE *mutants' are not possible. Bacterial density values (OD_600_) for *S*. Schwarzengrund were also similar to bacterial density values for the other serotypes. The differences in bioluminescence expression are due to a difference in host serotype background. Determination of the cause of this serotype-specific effect is beyond the scope of the current manuscript. It is of interest that expression of bioluminescence in *S*. Schwarzengrund was also the lowest in the plasmid *lux *system, pAK*lux*1, reported previously [19]. These results indicate plasmid pBEN276 can be utilized to construct a stable reporting system within the chromosome of *Salmonella enterica *serotypes for use in extended *in-vitro *and *in-vivo *trials.

### Assessment of bioluminescent assay at various temperatures

Genes *luxCDABE *encode bacterial luciferase which catalyzes the oxidation of reduced flavin mononucleotide (FMNH_2_) and a long chain aliphatic aldehyde in the presence of O_2 _to produce flavin mononucleotide (FMN) and acid with light emission. Because FMNH_2 _production is dependent on a functional electron transport chain, only metabolically active bacteria emit light [23]. Thus, BLI provides a sensitive real-time measurement of the effects of various chemical, biological and physical stimuli on bacterial metabolism [24]. We utilized our bioluminescent *Salmonella enterica *serotypes to validate our model under a temperature range that bacteria in food products are commonly exposed to (host to ambient to refrigeration). Therefore we investigated the relationship between cellular metabolic activity, characterized by bacterial light production, and temperature variation. The temperatures selected were 37°C, 25°C and 4°C.

Mesophiles, such as *Salmonella *grow best in moderate temperatures (15-40°C) with normal enzymatic activity. In this experiment luciferase reaction within *Salmonella *was monitored. At 37°C and 25°C BLI measurements were consistent within the replicates of the different serotypes. However, a change in temperature will have an impact on enzyme kinetics. Decreasing temperature, to 4°C, will slow molecular motion and inhibit the luciferase reaction. Decreasing temperature will also decrease the rate of metabolism, which translates to decreased concentration of substrate, FMNH_2_, available for the luciferase reaction. At 4°C we observed an expected reduction in bioluminescent signal compared to readings at the two higher temperatures, 37°C and 25°C (data not shown). However, over the time required (approximately 1 min) to complete BLI measurements at 25°C we observed a rapid increase in the bioluminescent signal between the first and the last wells read. We found that luciferase activity is restored shortly after removal from refrigeration temperature, so temperature effect is minimal after introduction to ambient temperatures (≥ 25°C). These results were consistent and validated that our reporting system using bioluminescent *Salmonella *can be successfully applied to monitor within a temperature range that bacteria in food products are commonly exposed to.

The stage on our luminometer has adjustable temperature with the lowest temperature setting being 25°C. Future work will include the development of a mechanism for maintaining plates at refrigeration temperatures while on the reading stage of the instrument to overcome this limitation.

### Development of chicken skin assay for real-time monitoring of bioluminescent *Salmonella enterica*

*Salmonella *presents a major problem for the poultry industry due to its persistence during the processing of chicken carcasses and few options exist that completely eliminate the bacteria from the chicken carcasses besides proper cooking. The physiological mechanisms which allow the bacteria to persist throughout processing of the chicken carcass are largely unknown and until the mechanics of attachment of the bacteria are more comprehensively understood, *Salmonella *may well remain a food safety concern.

**Figure 4:**
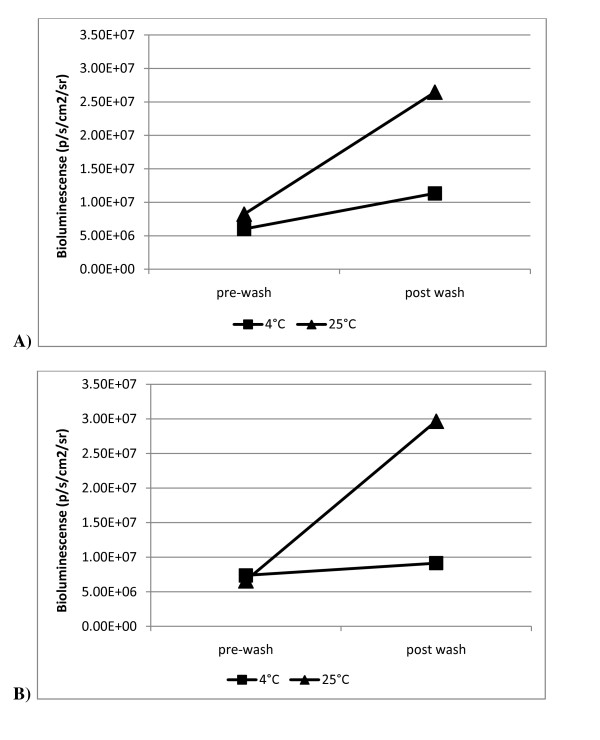
**Monitoring bacteria number following 25°C and 4°C water washes**. Bioluminescence quantified at 37°C before and after water washes at 4°C and 25°C. A) *S*. Mbandaka. B) *S*. Montevideo.

We have developed a model, which uses a real-time stable reporting system incorporating our bioluminescent tagged *Salmonella enterica *serotypes, which can be used to evaluate various pathogenic mitigation strategies. Further, this model may eventually aid in the understanding of how these serotypes are able to survive the processing continuum. We performed this experiment to demonstrate the potential value of this model as a screening tool by evaluating the performance of our bioluminescent *Salmonella *on chicken skin sections at two temperatures in an aqueous environment. We selected *S*. Mbandaka and *S*. Montevideo for this skin attachment experiment based on the consistent bioluminescence expression we observed within these serotypes (Figure 3). Individual aqueous solutions, each containing a *Salmonella enterica *serotype, were prepared and introduced to chicken skin according to protocol (described below). Separate plates (24-well) containing replicates of each serotype were placed on a rotating stage at 4°C and 25°C for 2 h. Immediately following this step, bioluminescent imaging was collected after a five minute interval at 37°C for both serotypes and is reported (Figure 4). Bioluminescent monitoring demonstrated the ability to quantify bacteria numbers on chicken skin following cold and warm washes. Our previous work showed washing with 25°C water suppressed the reproduction of *Salmonella *on chicken skin likely through the physical removal of bacteria [19]. Given that *Salmonella *is a mesophile, refrigeration temperatures further limit bacterial growth and the bacteria become metabolically static. Bioluminescent values, confirming bacteria numbers, at post-wash (4°C) were not shown to be significantly different compared to pre-wash values for both serotypes (*P *≥ 0.25). Bioluminescent values at post-wash (25°C) were greater compared to pre-wash values but the difference was not shown to be significantly different (*P *≥ 0.125). The increase in bioluminescence following the 25°C wash period is due to increased bacteria growth under favorable metabolic conditions (temperature) and nutrients provided by the chicken skin in solution. With our model we were able to quantify a change in bacteria number by monitoring bioluminescence following treatment.

These results provide evidence that our model may serve as an accurate and efficient means for *in-vitro *evaluation of the efficacy of pathogen mitigation strategies, i.e. antimicrobial compounds (AMC) and processing parameters, that may be utilized in the poultry processing industry to control *Salmonella enterica*. Future work utilizing our *lux *reporter system in our chicken skin model will feature an extended time course to better reflect the duration of exposure to conditions bacteria might be subjected to in the poultry processing environment.

## Conclusions

Our work demonstrates a novel, real-time monitoring system for *Salmonella enterica *serotypes that is stable and has potential use for in *in vivo *and *in vitro *trials. Our results show the efficiency of plasmid pBEN276 to confer bioluminescence to eleven wild-type *Salmonella enterica *isolates by inserting the *luxCDABE *operon into the *attTn7 *site on the chromosome. Chromosomal insertion of the gene is significant in that external antibiotic pressure is not required for perpetuation of the *luxCDABE *cassette. This system has the potential to eventually be utilized for the evaluation of potential pathogen mitigation strategies upon *Salmonella *under different environmental conditions over extended time courses, which was not previously possible due to limitations of plasmid-based reporter systems.

Detection was successful following metabolic inactivity due to refrigeration temperatures and results provide support for application of our model in trials simulating processing plant environmental conditions. Future experiments are planned using this system to evaluate the efficacy of various AMCs. We expect this research may provide a foundation for future work to understand the mechanism of attachment of *Salmonella *to chicken skin and its ability to persist during the poultry processing continuum.

## Methods

### Bacterial serotypes and growth media

As part of a previous study, *Salmonella enterica *isolates from five different sites along the broiler production continuum (day one placement, end of growout, arrival at the plant, pre-chill tank, and post-chill tank) were cataloged [25]. In the current study, 11 *Salmonella enterica *serotypes (*S*. Alachua*, S*. Braenderup*, S*. Enteritidis*, S*. Heidelberg*, S*. Kentucky*, S*. Mbandaka*, S*. Montevideo*, S*. Newport*, S*. Schwarzengrund*, S*. Seftenberg*, S*. Typhimurium) were selected. *Salmonella enterica *serotypes were cultured using Luria-Bertani broth and agar plates at 37°C. Ampicillin (100 μg mL^-1^) and was used for selection and 0.1% arabinose was used for transposition induction.

### Construction of plasmid pBEN276

The *luxCDABE *operon was amplified from the genome of *Photorhabdus luminescens *using primers PG131 (GATGCTACCTCGAGGTACAACCAGTTTGCAAGATG) and PG132 (TACGCTCAGGATCCGAATTCACTCCCTTGCCATC) and cloned in pCR2.1 (Invitrogen) to yield plasmid pBEN139. Primers PG131 and PG132 were added to include *Xho*I and *Bam*HI restriction sites. *A Xho*I-*Bam*HI restriction fragment from plasmid pBEN139 carrying *luxCDABE *was subcloned into plasmid pBEN129, a derivative of plasmid pACYC184 [26] containing *Xho*I and *Bam*HI sites, yielding plasmid pBEN135. A *Xho*I-*Not*I fragment from plasmid pBEN135 carrying the *luxCDABE *operon was subcloned into plasmid pGRG25 [20] to give plasmid pBEN275. The promoter of the housekeeping gene *frr *[27] was amplified from the *E. coli *K-12 MG1655 genome using PG209 (GTCTGACTCGAGGAATTCTTCCCGTGATGGATAAATAAG) and PG210 (CATCACTCGAGGTTACGAATCCTTGAAAACTTG) primers and cloned into the *Xho*I site of plasmid pBEN275 to give plasmid pBEN276.

### Insertion of *lux *genes into the chromosome of *Salmonella enterica*

Bioluminescence was established in the chromosome of the *Salmonella enterica *serotypes using plasmid pBEN276. The serotypes were grown to logarithmic phase (OD_600 _0.6-0.8), washed with 15% cold glycerol solution four times to make electrocompetent, and stored at -80°C. The serotypes were transformed with plasmid pBEN276 by electroporation using a Gene Pulser II system (Bio-Rad). Optimal electroporation conditions for *S*. Alachua, *S*. Heidelberg, *S*. Kentucky, *S*. Mbandaka, *S*. Newport and *S*. Seftenberg were 2.5 kV, 25 μF and 400Ω, and optimal conditions for *S*. Braenderup, *S*. Enteritidis, *S*. Montevideo, *S*. Schwarzengrund and *S*. Typhimurium were 1.8 kV, 25 μF and 600Ω. Bacteria were recovered for 1 h at 30°C in SOC media and then spread on LB plates with ampicillin and placed in an incubator at 30°C for approximately 16 h. Ampicillin resistant colonies were picked and cultured in LB broth with arabinose at 30°C for approximately 16 h to induce transposition. The cultures were streaked on LB agar and placed in an incubator at 42°C for approximately 16 h to cure the plasmid. Ten individual colonies were picked from this plate and cultured in LB broth at 42°C for approximately 16 h. Bioluminescent colonies were detected using a ChemiImager 5500 imaging system with AlphaEaseFC software (Alpha Innotech) or an IVIS Imaging System 100 Series with Living Image Software v2.50 (Xenogen). Bioluminescent cultures were subcloned in LB broth with ampicillin and placed in an incubator at 30°C for approximately 16 h. No visual evidence of growth confirmed absence of the plasmid.

### Characterizing the bioluminescent properties of *Salmonella enterica *serotypes

The bioluminescent *Salmonella enterica *serotypes were grown overnight in LB broth to reach stationary phase, and bacterial density value (OD_600_) of each serotype was determined in a 96-well clear-bottomed black cell culture plate (Costar) using ThermoMax spectrometer (Molecular Devices). Following bacterial density measurements, four separate dilution series were prepared for each serotype in 96-well clear-bottomed black cell culture plates. In each plate, the first four columns contained 10 fold dilutions (1.00 × 10^0 ^to 1.00 × 10^-3^), while the remaining eight wells contained doubling dilutions (5.00 × 10^-4^, 2.50 × 10^-4^, 1.25 × 10^-4^, 6.25 × 10^-4^, 3.13 × 10^-5^, 1.56 × 10^-5^, 7.81 × 10^-6^, 3.91 × 10^-6^, 1.95 × 10^-6^, 9.77 × 10^-7^, 4.88 × 10^-7^, 2.44 × 10^-7^). Bioluminescence was measured for 10 s of exposure using an IVIS Imaging System 100 Series, and bioluminescence was quantified using Living Image software v2.50. The last dilution of each series was spread on LB agar to determine the number of viable bacteria. The linear relationship between population densities and bioluminescence was determined by plotting bioluminescence against bacterial density as determined by plate counts and by linear regression analysis (PROC REG, SAS for Windows v9.2, SAS Institute Inc.). The minimum detectible number for each serotype was determined using the number of bacteria present in the last dilution that had detectable bioluminescence. Colony counts were also used to calculate the theoretical amount of bioluminescence produced from 1 CFU for each serotype.

### Transgene stability in the chromosome of *Salmonella enterica*

Transgene stability following insertion by plasmid pBEN276 in the eleven *Salmonella enterica *serotypes was analyzed by subcloning these bioluminescent *Salmonella enterica *serotypes in non selective LB broth every 24 h for a period of fourteen days. Technical replicates for each serotype were made in quadruplicate. For each passage, the previous culture was subcloned 1/10 the volume into new 300 μL cultures of LB broth in 96-well clear-bottomed black cell culture plates. Bacterial density and bioluminescence was measured at 12 h of growth at approximately every 3 days. Bioluminescence was measured using an IVIS Imaging System for 15 s of exposure and normalized by dividing total flux of bioluminescence by the corresponding bacterial density value. The average normalized bioluminescence for each serotype and passage was determined, which revealed the ability of each serotype to maintain the *lux *operon in its chromosome without antibiotic selection.

### Assessment of bioluminescent assay at various temperatures

An experimental model was established to investigate the relationship between temperature variation and metabolic activity, characterized by bioluminescence expression. Bioluminescence and bacterial density were measured using the LMax luminometer (1 s exposure time) and the Spectramax Plus 384 spectrophotometer (Molecular Devices), respectively. Cultures of bioluminescent *Salmonella enterica *serotypes were grown overnight (~16 h) to reach stationary phase and were diluted 10 fold with LB broth and 200 μL of the diluted bacteria suspension was inoculated into a 96-well clear-bottomed black cell culture plate and incubated at 37°C for 2 h to reach early log phase. Four technical replicates for each serotype were prepared. The initial bioluminescence and bacterial density reading was collected for the early log phase cultures at 37°C. Next, the plate incubated for 10 min at 25°C, and bioluminescence and bacterial density readings were measured. Then, the plate was transferred to 4°C and stayed at this temperature for 2 h, interrupted every 30 min to measure bioluminescence and bacterial density.

### Development of chicken skin assay for real-time monitoring of bioluminescent *Salmonella enterica*

Overnight cultures of bioluminescent *Salmonella enterica *serotypes, *S*. Mbandaka and *S*. Montevideo, were prepared in replicates in quadruplicate. Each individual solution was diluted to approximately 1 × 10^6 ^CFU/mL in distilled water, and 1 mL of the bacteria suspension was added to 8 mm circular chicken skin sections in 24-well polystyrene clear-bottomed black tissue culture plates (Wallac) as described previously [19]. Plates were incubated at room temperature (25°C) for 1 h to allow bacteria to attach to the skin. Following incubation, the suspension was vacuumed from each well, and skin sections were gently washed with distilled water and vacuumed to remove unattached bacteria. This washing process was repeated once more.

After removal of excess solution, initial bioluminescence on skin sections was quantified for 15 s of exposure using the IVIS imaging system. One mL of 4°C distilled water was added to each well of the appropriate plate for each serotype. The other plate for each serotype received one mL of 25°C distilled water. The plate that received 4°C distilled water remained at refrigeration temperature (4°C) for 2 h on a rotating stage at 200 rpm. The plate that received 25°C distilled water remained at room temperature (25°C) for 2 h on a rotating stage at 200 rpm. At the conclusion of the 2 h washing period, water was vacuumed from each well, and bioluminescence from bacteria attached to the chicken skin was measured at 37°C for 5 min. The total flux of bioluminescence from each well was divided by the corresponding bacterial density value of the original bacterial suspension to normalize bioluminescent flux.

## Authors' contributions

RB and RW isolated the *Salmonella *strains. PG constructed the pBEN276 plasmid. AK, RB, KH, and ML designed the bacteriological and genetic studies. AK, RW and KH performed the experiments and data analyses. AK, RB, KH, ML, RW and PG drafted the manuscript. All authors read and approved the final manuscript.
